# From motor primitives to context-dependent coordination: a system neuroscience approach integrating cognitive, affective, and experiential factors into muscle synergy analysis

**DOI:** 10.3389/fnsys.2026.1838867

**Published:** 2026-07-15

**Authors:** Alessandro Scano

**Affiliations:** Laboratory of Advanced Methods for Biomedical Signal and Image Processing, Institute of Intelligent Industrial Technologies and Systems for Advanced Manufacturing (STIIMA), Italian National Research Council (CNR), Milan, Italy

**Keywords:** muscle synergies, model, layer, experiential, motor control, affective, cognitive

## Abstract

Muscle synergies are traditionally viewed as stable, low-dimensional neuromuscular modules reflecting neural constraints. However, growing evidence suggests this mechanistic interpretation is incomplete. We propose a conceptual extension of the framework, arguing that coordination patterns reflected into multichannel EMG are emergent properties of an integrated cognitive–motor system rather than purely motor primitives. We identify three classes of non-motor factors that may shape motor output: (i) Task internalization and learning dynamics: synergy structure evolves with practice and the formation of internal models; (ii) affective and psychological states: emotional conditions (e.g., stress, anxiety) modulate muscle co-activation and stability; (iii) prior experience and sensorimotor memory: embodied history biases action selection and coordination. Consistent with predictive processing and embodied cognition, we argue these factors are constitutive dimensions of motor control rather than mere noise. This perspective implies that inter-subject variability in synergy structure reflects systematic differences in cognitive, affective, and experiential states. To enhance interpretability, we propose integrating minimal assessments into experimental designs: (i) task strategy evaluation, (ii) affective state characterization via physiological proxies or scales, (iii) documentation of prior motor experience, and (iv) analysis of learning trajectories. This integrative view complements existing models, providing a richer theoretical foundation to interpret variability, adaptability, and individual differences in human motor control.

## 1 Introduction

The control of human movement is characterized by a remarkable balance between flexibility and stability, enabling the generation of coordinated motor behavior across a wide range of tasks and contexts (Schmidt et al., 2018). One of the most influential frameworks proposed to account for this organization is the concept of muscle synergies, according to which the central nervous system simplifies motor control by recruiting groups of muscles as low-dimensional modules (Bizzi et al., 2002; d’Avella and Tresch, 2001; Tresch et al., 2006; d’Avella et al., 2003). Within this perspective, muscle activations can be approximated as combinations of a limited set of spatial patterns and their corresponding temporal coefficients, often formalized as EMG = c⋅ W, where *EMG* is the envelope achieved after processing multi-channel EMG, *W* are the spatial synergies and *c* the temporal activations. Empirical support for this framework has been reported across species, behaviors, and experimental paradigms, suggesting that such modular organization may reflect fundamental constraints in the neuromuscular system (d’Avella and Bizzi, 2005; Bizzi et al., 2008). Despite its success, the muscle synergy framework has been predominantly interpreted in mechanistic terms, with synergies viewed as relatively stable building blocks rooted in spinal or subcortical circuitry. This interpretation has been particularly influential in studies aiming to identify invariant structures of motor coordination across tasks or individuals. However, since preliminary intuitions that the body dimension cannot be separated from psyche, cognition and mind, a growing body of work in motor behavior and cognitive neuroscience suggests that motor output cannot be fully understood without considering the broader cognitive and affective context in which it is generated (Cromwell and Panksepp, 2011). Indeed, movement is not only the execution of motor commands but also the outcome of processes involving perception, prediction, evaluation, and decision-making, which is evident in child neuromotor learning (Shi and Feng, 2022). Contemporary theories such as Predictive Processing propose that the brain continuously generates and updates internal models to anticipate sensory consequences of action (Walsh et al., 2020; Hohwy, 2020), while frameworks of Embodied Cognition emphasize that cognition is deeply grounded in sensorimotor processes and bodily experience (Wilson and Golonka, 2013; Foglia and Wilson, 2013; Wilson, 2002). Within these perspectives, motor behavior emerges as a synthesis from the dynamic interaction between internal predictions, environmental constraints, and ongoing sensory feedback, rather than from the activation of isolated motor modules. Converging evidence further indicates that cognitive factors such as task representation, attention, and strategy selection, as well as affective states including stress and motivation, can significantly modulate motor performance and variability (Friston, 2012; Friston et al., 2011; Gallagher, 2020; Jeannerod, 1994). From a motor control standpoint, these observations raise a fundamental question: to what extent do muscle synergies reflect intrinsic properties of the motor system only, as opposed to emergent patterns shaped by higher-level cognitive and affective processes in relation with the motor system? While previous studies have acknowledged inter-subject (or even intra-subject) variability in synergy structure across individuals and contexts (Zhao et al., 2021; Pale et al., 2020), such variability is often treated as noise or secondary to the identification of canonical modules. This assumption may overlook the possibility that a portion of this variability is systematic and functionally meaningful or related to further cognitive, affective, or existential layers of control, as hypothesized for robot control (d’Avella et al., 2015). In this work, we argue that muscle synergies could be reinterpreted as context-dependent coordination patterns emerging from an integrated cognitive–motor system. Specifically, we propose that cognitive, affective, and experiential factors can perturb motor output, or even actively shape it by modulating the temporal structure of motor commands. Within the standard synergy formulation, this implies a selective influence on the activation coefficients (c), associated with cortical and premotor dynamics, while the structural components (W), often linked to lower-level circuitry, remain comparatively stable. While we acknowledge that extreme states or long-term adaptation might induce plasticity in W itself, we treat its stability as a first-order approximation to isolate and quantify the immediate impact of contextual variables on temporal control. Such a view provides a principled bridge between high-level cognitive processes and low-level motor execution, reframing motor variability as a signature of adaptive control rather than as an experimental artifact. It is important to note that the present framework is primarily conceptual in nature. Rather than introducing a fully specified computational model, we propose a meso-level interpretative architecture that bridges theoretical constructs from cognitive neuroscience with measurable motor variables. Thus, this manuscript is designed as a theoretical and methodological Perspective. Its primary goal is not to present a novel *in vivo* dataset, but to expose a critical analytical blind spot in standard synergy extraction methods and to establish a mathematically sound rationale for future experimental designs. The analytical formulations introduced in later sections should therefore be understood as an initial operationalization, intended to guide future empirical and computational developments rather than to provide a complete generative model. Building on this perspective, the present study aims to (i) articulate a conceptual framework that integrates muscle synergies with cognitive and affective layers of control, and (ii) outline methodological implications for the design and interpretation of motor control experiments. By situating muscle synergies within a broader cognitive–motor architecture, we seek to move beyond a strictly reductionist account and toward a more comprehensive understanding of how coordinated behavior is generated, modulated, and adapted across individuals and contexts.

## 2 Materials and methods

### 2.1 Conceptual framework: a cognitive–affective-experiential architecture of motor synergies

We propose a hierarchical yet dynamically coupled architecture in which motor behavior emerges from the interaction between cognitive, affective, and experiential processes, rather than from purely motor mechanisms. In this framework, motor output reflects the integration of multiple layers operating at different levels of abstraction, each contributing to the shaping of coordination patterns. At the highest level, the cognitive layer (internal task model) encompasses processes related to task representation, task understanding, and strategy selection, as well as attentional allocation and cognitive load. This layer defines how the task is internally encoded and constrains the space of possible motor solutions. Closely interacting with it, the affective layer (internal state modulation) includes emotional state, stress, fatigue, arousal, which globally modulate motor behavior by influencing factors such as muscle tone, variability, and stability. In parallel, the experiential layer (embodied prior) captures the contribution of prior motor experience, learned motor repertoires, and sensorimotor memory, providing a structured set of constraints that bias action selection based on past interactions with similar tasks. These higher-level layers converge onto the motor system, defined here as the combined neural and task space in which muscle activations, extracted muscle synergies (W), their temporal activations, and resulting kinematics are expressed. Within this system, observed motor output is treated as the emergent result of context-dependent modulation that includes the concepts of fixed motor primitives. Crucially, the architecture is not purely feedforward. While cognitive, affective, and experiential layers exert top-down influences on motor execution—collectively described as context-dependent shaping—the motor system, in turn, generates feedback signals that update higher-level representations. Motor performance influences the cognitive layer through the refinement of task representation, the affective layer through success -or failure- related emotional responses, and the experiential layer through learning and memory consolidation. This bidirectional coupling defines a continuous adaptive update loop, through which behavior evolves over time. In addition to vertical interactions, the model incorporates lateral coupling between layers. Cognitive and affective processes interact through mechanisms such as stress-dependent attentional modulation; cognitive and experiential layers interact through memory-driven strategy selection; and affective and experiential layers interact via emotionally weighted memory processes. These interactions impose *reciprocal constraints* that further shape motor output in a context-sensitive manner. Finally, we conceptualize the overall system as evolving within a cognitive–motor state space, in which behavior can be represented as a trajectory shaped by the continuous interaction of internal states and motor dynamics. Within this space, muscle synergies are not interpreted as fixed building blocks, but as emergent coordination patterns arising from the dynamic coupling of neural, cognitive, affective, and experiential processes.

### 2.2 Cognitive layer: internal task model and action selection

The cognitive layer encompasses the set of processes through which an individual construct an internal model of the task and selects an appropriate motor strategy. This includes task representation, task understanding, strategy selection, and the allocation of attentional resources. Within this layer, movement is not merely executed, but first conceptualized as a goal-directed action embedded within a specific context. A central function of this layer is the transformation of external instructions and environmental constraints into an internal representation that defines the space of admissible motor solutions. Importantly, this representation is not fixed: it depends on the subject’s interpretation of the task, prior knowledge, and current cognitive state. As a consequence, even when identical instructions are provided, different individuals may construct distinct internal models, leading to qualitatively different motor strategies. This aspect becomes particularly evident when contrasting simple and complex motor behaviors. In highly constrained or overlearned tasks, the cognitive layer may play a relatively limited role, with action selection becoming largely automatic. In contrast, in situations requiring strategic decision-making—such as a penalty kick in football—the cognitive layer becomes a dominant driver of motor behavior. In these contexts, the performer must integrate multiple sources of information, including opponent behavior, probabilistic expectations, and prior outcomes, to select not only how to execute a movement, but which movement to perform. Experimental evidence shows that such decision-making processes can significantly influence movement kinematics and timing, even when the final motor goal remains the same (Wolpert and Landy, 2012). From a theoretical standpoint, these processes are consistent with models of action selection and motor cognition proposed by researchers who emphasized the role of internal representations in linking perception and action (Gallese, 2000), and work on internal models highlights how the brain predicts and evaluates potential motor outcomes (Wolpert and Flanagan, 2001). Within the broader framework of Predictive Processing, the cognitive layer can be interpreted as generating high-level priors that constrain motor planning and guide the selection of action policies. Attention and cognitive load further modulate this layer by influencing the fidelity and stability of task representation. Under high cognitive load or divided attention, motor performance often becomes more variable and less efficient, reflecting a degradation in the precision of internal models. Conversely, focused attention can enhance the consistency of motor execution, although in some cases excessive conscious control may interfere with automatized skills, a phenomenon often described in the literature on “choking under pressure.” Crucially, within the present framework, the cognitive layer does not directly encode motor commands, but rather defines the structure of the control problem that the motor system must solve. In this sense, it acts as a high-level constraint on motor output, shaping the selection, timing, and coordination of muscle activity indirectly through its influence on downstream control processes.

### 2.3 Affective layer: internal state modulation of motor control

The affective layer encompasses the internal emotional and physiological state of the individual, including dimensions such as stress, anxiety, arousal, and motivation. Unlike the cognitive layer, which primarily defines the structure of the task, the affective layer acts as a global modulator of motor control, influencing how actions are executed under varying internal conditions. A substantial body of evidence demonstrates that emotional states systematically alter motor behavior. For instance, increased anxiety or stress is associated with elevated muscle co-contraction, reduced movement fluidity, and increased variability, reflecting a shift toward more rigid and conservative control strategies (Eifert and Forsyth, 2005). Conversely, positive or confident states are often linked to more efficient, flexible, and energetically economical motor patterns (Singer, 2002). These effects are not limited to gross motor behavior but extend to fine motor control, postural regulation, and timing precision. From a neurophysiological perspective, these influences are consistent with the role of neuromodulatory and limbic systems in shaping motor output (LeDoux, 2013). Within contemporary frameworks such as Predictive Processing, affective states can be interpreted as modulating the precision or gain of internal predictions, thereby influencing the balance between exploration and control in motor behavior. Importantly, the impact of the affective layer becomes particularly evident in performance-critical situations. In high-pressure contexts—such as competitive sports or skilled performance—emotional states can induce substantial changes in motor execution, even when the intended action remains unchanged. The phenomenon commonly referred to as “choking under pressure” exemplifies this interaction: heightened anxiety can disrupt well-learned motor patterns, leading to altered timing, increased stiffness, and degraded performance. Conversely, optimal arousal states can enhance performance by facilitating coordinated and adaptive motor output, in line with classical observations such as the Yerkes–Dodson law (Diamond et al., 2007). Within the present framework, the affective layer does not directly specify motor commands or task goals, but modulates the operating regime of the motor system. In particular, it influences parameters such as muscle tone, variability, responsiveness to feedback, and the stability of coordination patterns. As a result, the same cognitive representation of a task may give rise to markedly different motor outputs depending on the underlying affective state. Crucially, this modulation is not purely disruptive or noise-like. Rather, it reflects an adaptive mechanism through which the organism tunes motor behavior to internal and environmental demands. However, in experimental contexts, failure to account for affective state may lead to systematic biases in the interpretation of motor variability, particularly when comparing individuals or conditions with differing levels of stress or arousal.

### 2.4 Experiential layer: embodied priors and sensorimotor memory

The experiential layer captures the influence of prior motor experience, learned repertoires, and sensorimotor memory on the generation of motor behavior. Within this layer, action is shaped not only by current goals and internal states, but also by the history of interactions that the individual has had with similar tasks and environments. These accumulated experiences define a set of embodied priors that constrain and bias motor execution (Friston, 2011). A key feature of this layer is that prior experience does not merely facilitate performance in a generic sense, but actively structures the space of possible motor solutions. Individuals with different motor histories—such as athletes trained in distinct disciplines—often exhibit systematically different coordination patterns when performing superficially similar tasks (Schmidt et al., 2011). For example, a movement involving trunk rotation or upper-limb coordination may be executed using distinct muscle recruitment strategies depending on whether the subject’s background is, for instance, in throwing sports, racket sports, or non-athletic contexts. These differences cannot be fully explained by biomechanics alone, but reflect the reuse and adaptation of previously acquired motor patterns. This perspective is consistent with classical and contemporary accounts of motor learning and control. Early work on motor schemas and generalized motor programs emphasized how repeated practice leads to the formation of structured representations that can be flexibly adapted across contexts. More recent approaches highlight the role of experience-dependent plasticity in shaping sensorimotor mappings and action selection (Kiefer et al., 2007; Tosoni et al., 2008). Within the broader framework of Embodied Cognition, these stored motor patterns are not abstract representations, but are deeply grounded in bodily interaction with the environment. Importantly, the concept of “motor primitives” can be reinterpreted within this layer. Rather than being fixed, hardwired building blocks, primitives may reflect stabilized patterns of coordination that emerge through repeated experience and are subsequently reused across tasks. In this sense, what is often identified as a synergy or module may partially reflect the subject’s motor history, rather than a universal organizational principle of the motor system. This reinterpretation provides a potential explanation for inter-individual variability in synergy structure, suggesting that differences in extracted modules may arise from differences in learned repertoires. The influence of the experiential layer extends beyond the selection of spatial patterns of muscle activation to include temporal and dynamical aspects of movement. Well-learned actions are typically characterized by more stable timing, reduced variability, and increased efficiency, reflecting the consolidation of sensorimotor mappings. Conversely, unfamiliar tasks often elicit exploratory behavior, increased variability, and less stable coordination patterns, as the system searches for effective solutions within the available motor space. Within the present framework, the experiential layer acts as a structured prior that interacts with both cognitive and affective processes. It biases strategy selection, constrains the range of feasible motor solutions, and shapes how actions are adapted over time. Crucially, it provides a bridge between short-term modulation and long-term adaptation, linking immediate motor behavior to the accumulation of experience across trials and contexts.

### 2.5 Neural–motor layer: synergies, activation dynamics, and task-space output

At the lowest level of the proposed architecture, the neural–motor layer encompasses the mechanisms through which motor commands are translated into muscle activations and movement. This layer includes the neural processes underlying motor execution, the organization of muscle coordination patterns, and their expression in task space through kinematics and force production. Within the muscle synergy framework, this level is commonly described by representing muscle activity as a combination of spatial modules and their corresponding temporal activations, typically formalized as EMG = c⋅ W. In this formulation, the matrix W represents the structure of muscle synergies, often interpreted as groups of muscles that are co-activated as functional units, while the coefficients *c* capture their temporal recruitment across time. A substantial body of experimental work has demonstrated that a limited number of such components can account for a large proportion of variance in electromyographic signals across a wide range of motor tasks, supporting the notion of a low-dimensional organization of motor control. From a neurophysiological perspective, muscle synergies have often been associated with spinal or subcortical circuitry, where convergent neural pathways may give rise to coordinated activation patterns across multiple muscles. At the same time, cortical and premotor areas are known to contribute to the selection, timing, and modulation of these patterns, suggesting a hierarchical organization in which higher-level structures influence the recruitment of lower-level modules. This view is consistent with evidence from both animal and human studies indicating that motor cortex activity is closely linked to the temporal structure and scaling of muscle activation, rather than solely to the specification of individual muscles. In addition to muscle activations, this layer includes the mapping from neural commands to observable behavior in task space, such as joint kinematics, endpoint trajectories, and force production. Importantly, similar task-level outcomes can often be achieved through multiple combinations of muscle activations, reflecting the well-known redundancy of the motor system. Within the synergy framework, this redundancy is partially resolved by constraining control to a lower-dimensional subspace, while still allowing flexibility in the generation of behavior. Despite the robustness of this framework, an open question concerns the extent to which the extracted components W and c reflect intrinsic properties of the motor system as opposed to context-dependent adaptations. While W is often treated as relatively stable across tasks and conditions (in the spatial synergy framework), the temporal coefficients c are inherently dynamic and sensitive to task demands, timing constraints, and external perturbations. This distinction suggests that different elements of the synergy model may be differentially influenced by upstream processes. Within the present architecture, the neural–motor layer is therefore treated as the locus where higher-level constraints are instantiated into concrete motor output. Rather than being an isolated system, it operates under continuous modulation from cognitive, affective, and experiential layers, which shape the selection, timing, and coordination of muscle activity. In this sense, the observed motor output—and the synergies derived from it—are interpreted as emergent patterns arising from the interaction between neural mechanisms and higher-level control processes, rather than as fixed building blocks of movement.

### 2.6 Inter-layer coupling: context-dependent shaping and adaptive feedback

**FIGURE 1:**
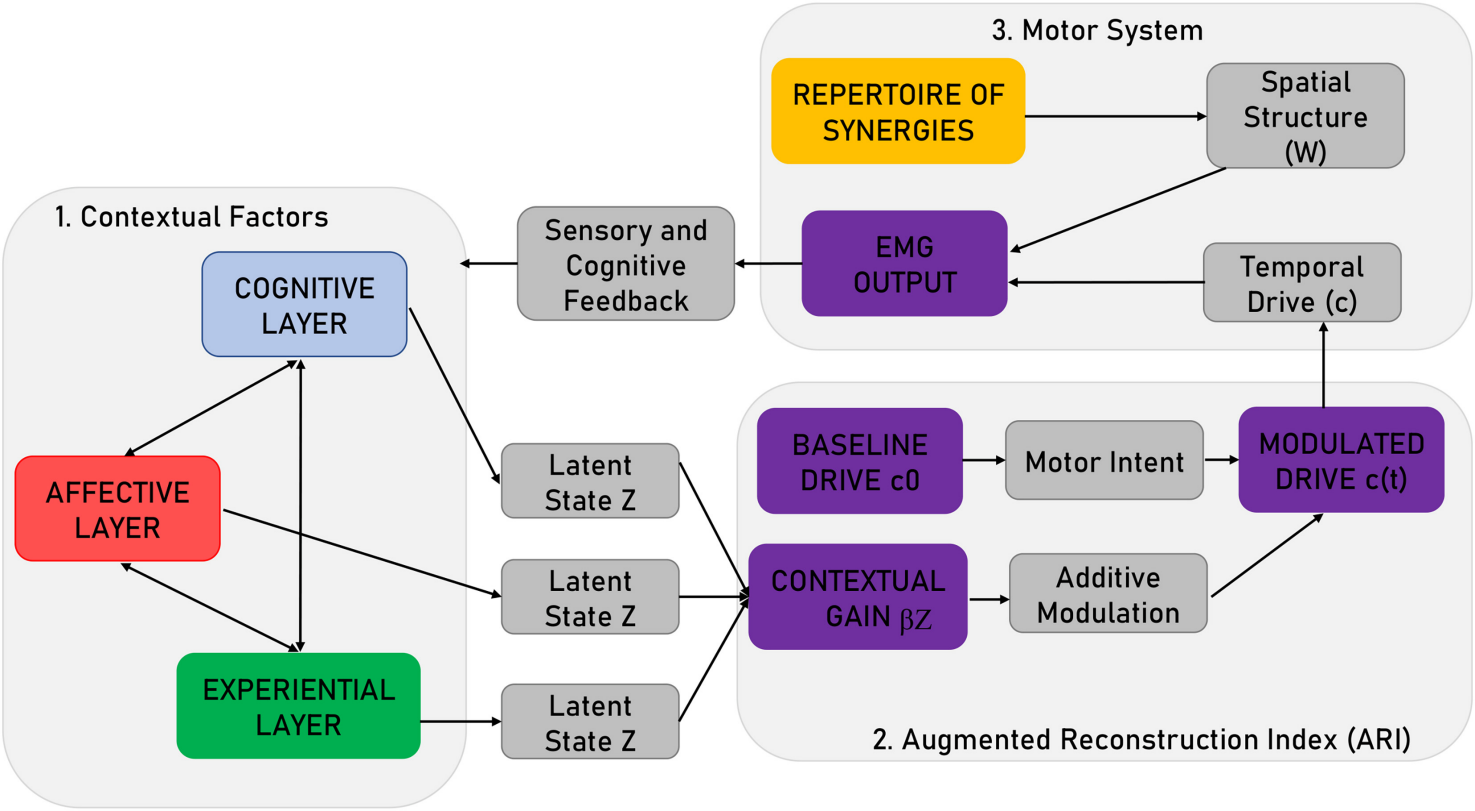
Architectural workflow of the Augmented Reconstruction Index (ARI) framework. The diagram illustrates the hierarchical integration of non-motor latent factors into the muscle synergy extraction process. 1. Contextual Factors and State Vector (Z): The top-level input represents the multi-dimensional latent state Z, emerging from the interaction of three primary layers: the Cognitive Layer (task representation, attentional load, and goal-directed intent), the Affective Layer (stress, arousal, and emotional valence), and the Experiential Layer (motor priors, embodied memory, and expertise). These factors constitute the contextual variance often ignored in standard decomposition models. 2. ARI Integration Logic: The framework decouples the observed temporal activation into two distinct components. The Baseline Drive (c_0_) represents the pure feedforward motor plan associated with the task under neutral conditions. The Contextual Gain (β⋅Z) quantifies the influence of the latent state Z on the motor output, scaled by the sensitivity vector β. These components are integrated to form the Modulated Coefficient c(t), defined as c(t) = c_0_ + β⋅Z, which serves as the final temporal command sent to the lower-level modules. 3. Motor Execution and Output: At the execution level, the modulated temporal drive c(t) recruits Stable Synergy Modules (W), which represent the hard-wired or long-term consolidated spinal primitives. The interaction between the spatial structure W and the context-dependent temporal drive c(t) generates the Final EMG Output. Feedback Loops: The model incorporates an adaptive closed-loop structure where the motor performance and sensory consequences are fed back to the higher-level layers. This allows for the dynamic updating of task models (cognitive), emotional state regulation (affective), and the consolidation of new motor patterns (experiential/learning). The ARI framework quantifies Cognitive–Affective Contributions to Motor Synergies. To move beyond a purely conceptual account, the proposed framework requires the integration of measurable proxies for cognitive, affective, and experiential variables within motor control experiments. While these dimensions cannot be directly observed, they can be approximated through a combination of behavioral, psychometric, and computational measures, enabling a principled extension of synergy-based analyses. Importantly, our aim is to promote the role of cognitive-affective-experiential assessment when extracting muscle synergies, and to suggest possible implementation compatible with standard purely motor approaches, even if a detailed model of cognitive-affective-experiential factors is beyond the scope of the work. A formal mathematical definition is provided in sections 2.10–3.4.

The proposed architecture is defined not only by the presence of distinct functional layers, but by the structured interactions that dynamically couple them into a coherent control system. Rather than reiterating the role of each layer, this section focuses on the mechanisms through which their interaction gives rise to adaptive motor behavior (Table 1). A first key mechanism is context-dependent shaping, whereby higher-level processes modulate motor output through complementary pathways. Instead of acting independently, cognitive, affective, and experiential variables jointly constrain the space of possible motor solutions. Their combined influence results in a context-sensitive modulation of coordination patterns, such that identical task demands can lead to systematically different motor outputs depending on the internal configuration of the system. Crucially, this modulation does not operate in a strictly hierarchical or feedforward manner. The architecture incorporates closed-loop feedback dynamics, in which motor execution continuously informs higher-level processes. Performance-related signals contribute to the refinement of task representation, the updating of internal state, and the consolidation of experience. Through this recursive process, behavior is progressively adapted across trials and contexts. In addition to vertical interactions, the system is characterized by lateral dependencies between higher-level processes. Cognitive, affective, and experiential factors do not act as separable dimensions, but mutually constrain one another. For instance, internal representations of the task may be biased by prior experience, while affective state can influence both attentional allocation and strategy selection. These interdependencies further contribute to shaping motor output in a history- and state-dependent manner. Within this integrated framework, motor behavior can be interpreted as the evolution of a trajectory within a cognitive–motor state space, where each state reflects a specific configuration of interacting processes. From this perspective, muscle synergies are not fixed entities, but emergent coordination patterns reflecting the instantaneous coupling between layers. Importantly, this view provides a principled reinterpretation of variability. Differences in synergy structure across individuals or conditions are not treated as residual noise, but as signatures of distinct internal states and interaction dynamics within the system (see Figure 1).

**TABLE 1:** Inter-layer connections and functional roles contribute to the shaping of the neuromotor system.

Input layer	Output layer	Type of connection	Functional role
Cognitive	Motor	Feedforward	Defines task goals, representation, and strategy; constrains solution space
Affective	Motor	Feedforward	Modulates gain, variability, stability, and muscle tone
Experiential	Motor	Feedforward	Provides embodied priors; biases coordination patterns and synergy recruitment
Motor	Cognitive	Feedback	Updates task representation based on performance and error
Motor	Affective	Feedback	Generates emotion-related responses (e.g., success/failure, confidence)
Motor	Experiential	Feedback	Drives learning and consolidation of motor memory
Cognitive/affective		Lateral (bidirectional)	Attention–emotion interaction; stress-dependent modulation of cognition
Cognitive/experiential		Lateral (bidirectional)	Memory-guided strategy selection; task reinterpretation based on experience
Affective/experiential		Lateral (bidirectional)	Emotionally weighted memory and experience-dependent modulation

### 2.7 Quantifying the cognitive layer

The cognitive layer can be operationalized by assessing how participants internally represent and solve the task. This can be approached at multiple levels. First, task representation and strategy can be probed through structured self-reports collected immediately after task execution, for instance by asking participants to describe the strategy they adopted or the features of the task they focused on. While subjective, these reports can reveal systematic differences in internal models across individuals. Second, attention and cognitive load can be quantified using established paradigms such as dual-task interference (Pashler, 1994), EEG techniques (Mastropietro et al., 2023), or validated psychometric instruments (Leppink et al., 2013). Variations in performance under cognitive load provide indirect evidence of the degree to which the task relies on active cognitive control. Third, the structure of motor planning can be inferred through comparison with optimal control models. For example, deviations from trajectories predicted by minimum-jerk or minimum-torque criteria can be used as indicators of alternative planning strategies, suggesting differences in how the task is internally represented.

### 2.8 Quantifying the affective layer

The affective layer can be characterized through a combination of subjective and physiological measures. Self-reported measures of emotional state, such as state anxiety or perceived stress, provide a direct but coarse estimate of internal conditions. Standardized instruments widely used in experimental settings include the State–Trait Anxiety Inventory (STAI; Spielberger et al., 1983) and the Perceived Stress Scale (PSS; Cohen et al., 1983), which allow a reliable quantification of transient and baseline affective states. These measures can be complemented by physiological proxies, including heart rate variability (HRV), skin conductance (electrodermal activity, EDA), and respiration patterns, which offer continuous and objective indices of autonomic arousal. HRV, in particular, is a well-established marker of stress-related autonomic regulation (Shaffer and Ginsberg, 2017), while EDA provides a sensitive measure of sympathetic activation (Boucsein, 2012). These signals can be temporally aligned with motor execution, allowing the investigation of how fluctuations in affective state correlate with changes in muscle activation patterns, co-contraction, and variability. Such multimodal approaches enable the identification of systematic relationships between emotional state and motor coordination. However, a critical methodological challenge in integrating these variables is the profound mismatch in temporal resolution between high-frequency motor data (EMG, kinematics) and sparse psychometric sampling. Furthermore, repeatedly administering lengthy questionnaires (e.g., the 40-item STAI) during an active motor task would impose an unacceptable cognitive burden, potentially inducing the very stress it aims to measure and artificially altering the synergy structure. To avoid this, we recommend a two-tiered operational approach. First, extensive psychometric instruments (STAI, PSS) should be utilized exclusively as pre- and post-session baseline anchors. Second, to capture intra-session dynamics without disrupting the motor flow, researchers should rely on continuous, non-invasive physiological proxies (HRV, EDA) recorded synchronously with EMG. When subjective self-reports are strictly necessary between experimental blocks, they should be implemented as ultra-brief Visual Analogue Scales (VAS) or single-item Ecological Momentary Assessments (e.g., a 1–10 digital slider for perceived effort or anxiety), requiring merely a few seconds to complete.

### 2.9 Quantifying the experiential layer

The experiential layer can be operationalized by capturing both long-term and short-term aspects of motor experience. Long-term experience can be assessed through structured questionnaires documenting participants’ motor history (e.g., sports practice, training background), allowing grouping or continuous characterization of subjects based on prior exposure. Short-term experience can be quantified by analyzing learning dynamics within the experiment, for instance through trial-by-trial changes in performance, variability, or synergy structure. Metrics such as adaptation rate, error reduction, and stabilization of coordination patterns provide indirect access to the updating of sensorimotor memory. Also, the subject could be asked whether the experimental gestures reminded of previously performed activities. Additionally, variability structure itself can be informative: more experienced individuals often exhibit structured variability aligned with task goals, whereas novices display more exploratory or unstructured patterns.

To synthesize the operationalization of these layers, Table 2 provides a structured list of candidate features and metrics that can be extracted to quantify the cognitive, affective, and experiential dimensions during motor tasks. These variables serve as the experimental input space for the dimensionality reduction techniques (e.g., PCA) used to estimate the latent contextual vector Z.

**TABLE 2:** Candidate metrics and features for the operationalization of contextual layers.

Contextual dimension	Assessment domain	Candidate metrics and features
Cognitive (task representation)	Psychometric and behavioral	Subjective task-complexity ratings (e.g., NASA-TLX); trial-by-trial strategic switches; ultra-brief Visual Analog Scales (VAS) for cognitive effort; dual-task interference (reaction times).
Affective (internal state)	Physiological and self-report	Continuous autonomic metrics (Heart Rate Variability—HRV, Electrodermal Activity—EDA, tonic pupil dilation); ultra-brief anxiety or arousal self-reports (VAS).
Experiential (embodied priors)	Historical and kinematic	Number of practice trials (learning curve attenuation); retention indices; expert vs. novice benchmarking coefficients; documented years of specific motor training; current profession/practiced activities.

### 2.10 Linking cognitive–affective-experiential variables to synergy structure

To integrate these measurements within the muscle synergy framework, we propose extending standard reconstruction-based analyses. Model quality is typically evaluated through the coefficient of determination R^2^_*motor*_, which reflects how well the extracted synergies reconstruct the observed signal. However, this approach does not account for variability arising from cognitive, affective, or experiential factors which may systematically modulate motor output without being captured by the synergy decomposition. To address this limitation, we propose to incorporate context-related variables. Rather than introducing a single scalar index, we define the Augmented Reconstruction Index (ARI) as a structured decomposition of explained variance:


$$\text{ARI = }\left({\text{R}}^{2}{}_{\text{motor}},\text{Δ}{\text{R}}^{2}{}_{\text{context}}\right)$$

where R^2^_motor_ captures the contribution of the synergy-based model, and ΔR^2^_context_ represents the additional variance explained by contextual variables, conditioned on the motor structure. This formulation preserves the interpretability of classical reconstruction metrics while explicitly quantifying the contribution of higher-level factors.

### 2.11 Interpretation

Within this framework, R^2^_motor_ captures the contribution of low-dimensional motor structure, while ΔR^2^_context_ quantifies the extent to which higher-level factors explain additional variance in motor output. Crucially, this formulation does not assume that cognitive–affective variables replace the synergy model, but rather that they modulate its expression, primarily through the temporal activation coefficients c(t). As such, the proposed approach provides a quantitative bridge between neural–motor mechanisms and higher-level processes, enabling a more complete interpretation of variability in motor coordination.

### 2.12 Computational proof-of-concept: simulation parameters

To evaluate the algorithmic behavior of the proposed framework, we implemented a bio-inspired toy model simulating an 8-muscle upper limb reaching task across 8 planar directions. The simulation parameters were set as follows:

Spatial modules (W): Three ground-truth spatial synergies were defined, unit-normalized to isolate amplitude fluctuations to the temporal domain.

Temporal drive (activations) (c_0_): Baseline temporal profiles were modeled as asymmetric biphasic bursts (acceleration/deceleration phases), with a 1% stochastic gain variation simulating natural neuromuscular jitter.

Contextual modulation: A tonic contextual state was simulated using a fixed latent variable *Z* = 0.6. The sensitivity vector was set to β = [0.175, 0.125, 0.15], introducing an asymmetrical tonic shift across the three modules to mimic realistic, module-specific susceptibility to stress.

## 3 Results

### 3.1 Analytical framework: integrating contextual variables into synergy-based models

To quantitatively account for the influence of cognitive, affective, and experiential factors on motor coordination, we extend the classical muscle synergy framework by explicitly incorporating context-dependent variables into the modeling of muscle activity. The following analytical formulation represents a first step toward the computational operationalization of the proposed framework. It is not intended as a complete or definitive model, but rather as a simplified and tractable approximation that captures key aspects of the interaction between motor structure and contextual variables.

### 3.2 Classical synergy formulation

Motor output is typically described as a low-dimensional combination of muscle synergies:


EMG⁢(t)=c⁢(t)⋅W+ϵ⁢(t) (1)

where W represents the spatial structure of muscle synergies, c(t) their temporal activation coefficients, and ϵ(t) the residual error. Model quality is commonly assessed through the coefficient of determination R^2^_motor_, which quantifies the proportion of variance in the EMG signal explained by the synergy decomposition. While effective in capturing the low-dimensional structure of motor output, this formulation does not account for variability arising from differences in internal state across subjects or trials.

### 3.3 Contextual modulation of temporal dynamics

Building on the proposed architecture, we introduce a vector of internal state variables (Equation 2):


Z=[Z,cZ,aZ]e (2)

For the practical implementation of the ARI framework, the latent contextual vector Z is not an unknown hidden variable to be mathematically inferred from the EMG signals. Rather, Z acts as a known empirical input (a covariate). It is continuously constructed and synchronized with the EMG using the two-tiered physiological assessment approach (e.g., via HRV or EDA signals) detailed in section 2.8. By providing Z as a known temporal constraint, the mathematical complexity of the model is significantly reduced. Z represents cognitive, affective, and experiential factors, respectively. To account for the heterogeneous nature and different time scales of the variables in Z (e.g., tonic physiological signals vs. psychometric scores), we define Z as a vector of latent factors extracted through dimensionality reduction techniques. Formally, let Z_raw_ be the set of collected non-motor metrics; the operational vector Z is obtained as Z = φ(Z_raw_) where φ represents a transformation (such as Principal Component Analysis or Factor Analysis) that standardizes the features and reduces collinearity. This ensures that the coefficient β (Equation 3) reflects the weight of integrated “internal states” rather than being biased by the different magnitudes of individual sensors. Aware of the limitations of this approach that constrains the analysis to the sole spatial module, we hypothesize that these variables primarily influence the temporal structure of motor commands, rather than the underlying spatial modules. This simplification is intended as a necessary step for the present operationalization, allowing for a tractable decomposition of variance. However, it establishes a baseline for future models that may incorporate context-dependent structural reorganization of the synergies themselves. Accordingly, we model the activation coefficients as:


c(t)=c(t)0+β⋅Z+η(t) (3)

where c_0_(t) represents the baseline activation pattern, β is a “sensitivity” coefficient that captures the entity of the influence of contextual variables, and η(t) denotes in general residual variability. Here, we put η(t) = 0 as noise is considered as a unique term in Equation 1 for simplicity.

Substituting into the original formulation yields an extended model (Equation 4):


EMG⁢(t)=c⁢(t,Z)⋅W+ϵ⁢(t) (4)

which explicitly links motor output to both synergy structure and internal state.

### 3.4 Variance decomposition and augmented reconstruction index

To quantify the contribution of contextual variables, we adopt a variance-partitioning approach. Let R^2^_motor_ denote the variance explained by the classical synergy model, and R^2^_ motor+context_ the variance explained by the extended model including contextual predictors. The additional contribution of contextual variables is defined as the incremental (semi-partial) coefficient of determination (Equation 5):


$$\Delta {\text{R}}^{\text{2}}{}_{\text{context}}\;\text{=}\;{\text{R}}^{\text{2}}{}_{\text{motor}\text{+}\text{context}}{\text{−R}}^{\text{2}}{}_{\text{motor}}$$ (5)

Rather than collapsing these quantities into a single scalar metric, we define the Augmented Reconstruction Index (ARI) as a vector of complementary components (Equation 6):


$$\text{ARI = }\left({\text{R}}^{2}{}_{\text{motor}},\text{Δ}{\text{R}}^{2}{}_{\text{context}}\right)$$ (6)

This representation explicitly separates the contribution of intrinsic motor structure from context-dependent modulation, enabling a more interpretable decomposition of motor variability.

### 3.5 Experimental and analytical pipeline

The proposed framework can be implemented within a standard experimental pipeline that integrates motor recordings with measurements of internal state. First, task design and experimental conditions define the external constraints under which behavior is generated. In parallel, cognitive, affective, and experiential variables are quantified using complementary approaches. Cognitive factors can be assessed through strategy reports, task understanding probes, or dual-task paradigms. Affective state can be measured using self-report questionnaires and physiological signals such as heart rate variability or skin conductance. Experiential factors can be characterized through participant history and through learning dynamics observed across trials. Motor output is then acquired through electromyographic recordings and kinematic measurements. Muscle synergies are extracted using standard decomposition techniques, yielding estimates of W and c(t). Subsequently, two models are constructed: a classical synergy model based solely on W and c(t), and an extended model in which contextual variables Z are incorporated as predictors of temporal activation patterns. Regarding contextual data pre-processing, we suggest that before integration with EMG data, all variables in Z undergo Z-score normalization to ensure zero mean and unit variance. Given the potential redundancy between physiological measures (e.g., heart rate and skin conductance both reflecting autonomic arousal), a Principal Component Analysis, a Factor Analysis or an Autoencoder can be applied. Only the components explaining a significant portion of the variance (e.g., > 80%) are retained. This step mitigates the “curse of dimensionality” and prevents overfitting in the subsequent ARI calculation. Model comparison is performed using the metrics defined above, allowing the quantification of both motor and context-dependent contributions to observed variability. Finally, performance-related feedback can be used to track adaptation over time, linking changes in motor output to updates in experiential and affective states. This enables the analysis of behavior not only as a static output, but as a trajectory evolving within a cognitive–motor state space.

### 3.6 Computational proof-of-concept: synergies vs. contextual modulation

(i) To illustrate the methodological advantage of the Augmented Reconstruction Index (ARI) over standard unconstrained factorization techniques, we developed a computational toy model. The goal of the simulation is not to reproduce physiological reality, but to demonstrate an identifiability failure in standard decomposition methods. We compared two simulated scenarios: Baseline Condition (“Relaxed” state): EMG activity was generated using the ground-truth spatial synergies and temporal activations according to (Equation 7):


EMG=basec⋅ground_truthW+ground_truthε (7)

Standard Non-negative Matrix Factorization (NMF) was first applied to this baseline dataset to estimate the reference spatial synergies (W_ground_truth_) and temporal coefficients c_ground_truth_.

(ii) Contextualized Condition (“High Stress” or Cognitive Load): the identical kinematic goal was pursued, but a simulated internal state variable Z introduced a context-dependent tonic modulation through a sensitivity vector β, such that (Equation 8):


c=contextc+ground_truthβ⋅Z (8)

It should be noted that the linear formulation adopted for the contextual modulation term (β⋅Z) represents a first-order approximation implemented for mathematical tractability. While neurophysiological interactions between central psychological states and peripheral motor outputs are known to exhibit highly non-linear behaviors, a linear additive model serves as a necessary baseline. This simplifying assumption isolates the core algorithmic question: demonstrating how even a straightforward, constant tonic perturbation can induce an apparent structural reorganization in unconstrained factorization methods (Equation 9).


EMG=contextc⋅contextW+ground_truthε

The contextualized dataset was then analyzed using two alternative approaches:

(i) standard unconstrained NMF, in which both W and c were freely estimated from the contextualized EMG data;

(ii) the proposed ARI framework, in which the spatial synergies were constrained to the baseline structure (W = W_ground_truth_), while contextual modulation was explicitly modeled through the β⋅Z term acting on the temporal coefficients.

**FIGURE 2:**
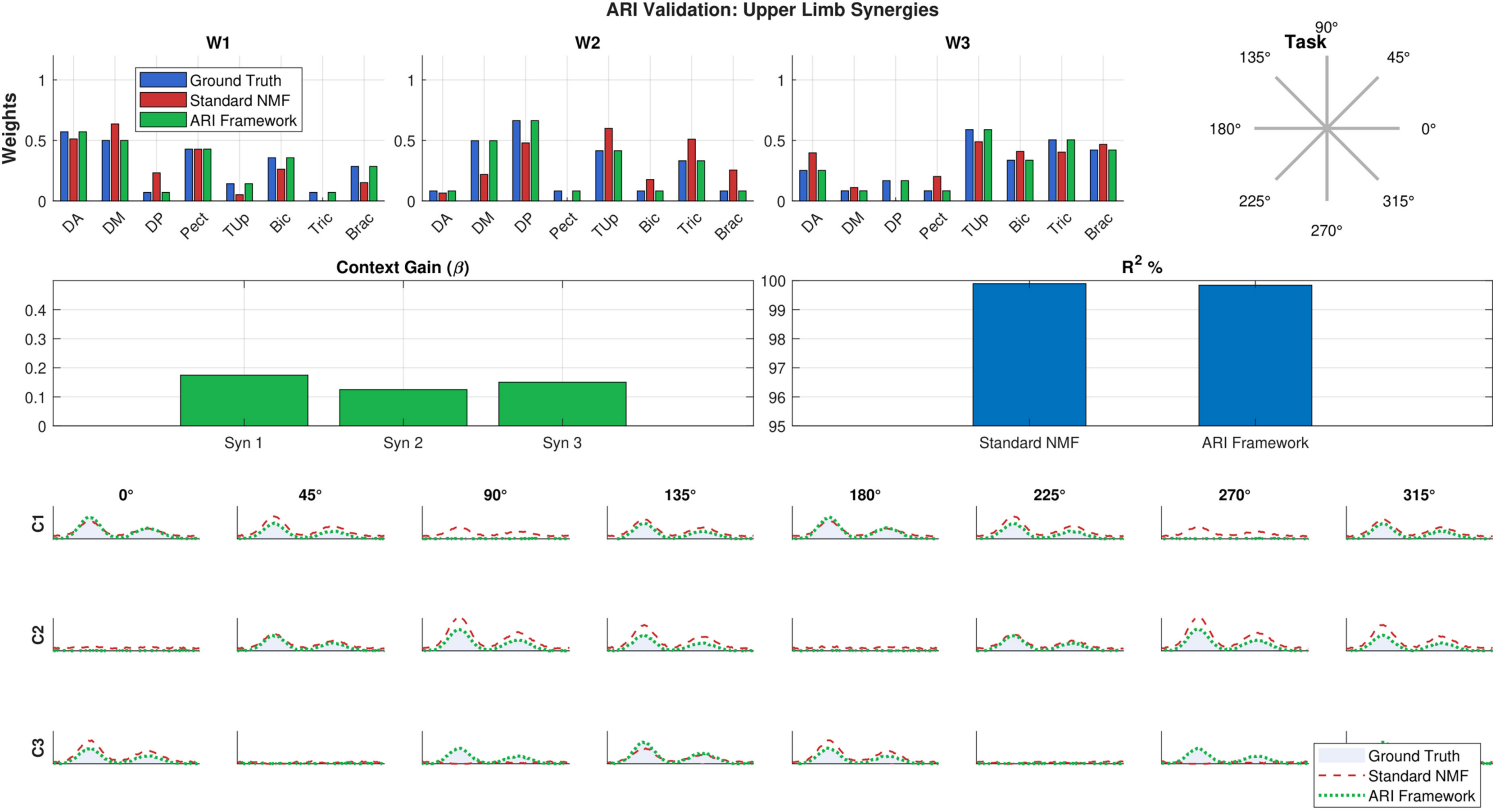
Validation of the Augmented Reconstruction Index (ARI) framework using a bio-inspired toy model of an 8-muscle upper limb reaching task. Top panel: Spatial Synergy Analysis: Ground Truth spatial synergies (blue) representing the original neuromuscular coordination in a “Relax” (baseline) condition. Synergies extracted via standard Non-negative Matrix Factorization (NMF) under “Stress” conditions (*Z* = 0.6) using unconstrained decomposition of the contextualized dataset are depicted in red. Note the structural drift: the NMF algorithm distorts the muscle weights to account for the tonic offset induced by contextual factors. In green, ARI framework reconstruction obtained by constraining the spatial synergies to the baseline structure while modeling contextual modulation separately: by anchoring the analysis to the baseline motor primitives, the original coordination structure is preserved, identifying the contextual influence as a separate component. Top-right panel depicts the simulation setup and motor task, consisting in the center-out point-to-point movements in 8 planar directions (0–315°). Middle panel (left): depicts the contextual gain vector β used in the simulation. Middle panel (right): R^2^ comparison when extracting three synergies; both methods achieve high reconstruction quality ( > 99%, NMF = 0.9989; ARI = 0.9983), but standard NMF reaches this through mathematical overfitting of muscle weights, while ARI achieves it through physiological modeling of contextual factors. Lower panel: Temporal Synergy Dynamics: Reconstructed activation coefficients c(t) across all 8 directions. The biphasic profiles (acceleration/deceleration) are directionally tuned. Standard NMF (red dashed line) consistently overestimates the baseline activation due to the absorption of the tonic stress component (Z). Conversely, the ARI reconstruction (green dotted line) effectively decouples the motor command from the contextual noise, overlapping with the Ground Truth “Relax” profile (light blue area). This demonstrates that the ARI framework prevents “coefficient pollution,” allowing for a cleaner isolation of the pure motor drive even in high-load or stressful contexts.

**FIGURE 3:**
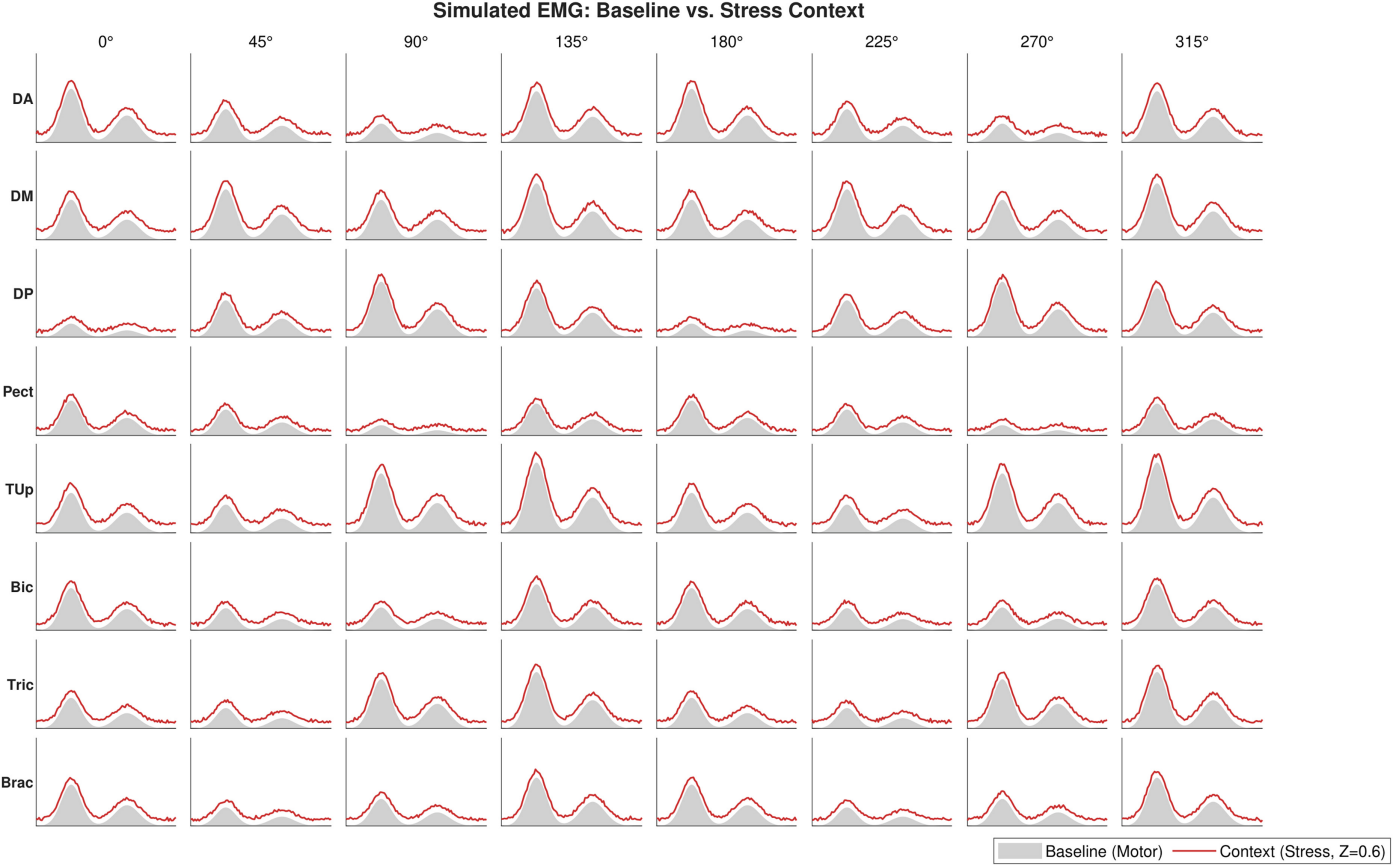
Simulated EMG traces. A representative comparison of the simulated EMG signal for a single muscle channel. The Baseline Motor condition exhibits standard phasic bursts returning to a zero-activation baseline. In contrast, the “Stress” condition (*Z* = 0.6) demonstrates how the modeled contextual modulation (β⋅Z) realistically introduces a sustained tonic elevation of the baseline and continuous low-level co-contraction, mirroring human physiological responses to cognitive and emotional load. A standardized, two-burst temporal profile was mapped across muscles to clearly illustrate the additive effect of the contextual matrix without loss of generality.

From a physiological perspective, the simulated contextualized EMG traces (EMG_context_) effectively mirror real-world electromyographic alterations observed under cognitive load or high-arousal states. The addition of the β⋅Z term generates a sustained elevation in the baseline EMG amplitude beneath the dynamic bursts. This pattern realistically reflects the increased generalized muscle stiffness, heightened sympathetic tone, and widespread low-level co-contraction typically exhibited by subjects performing motor tasks under psychological pressure, cognitive load, or stress. Extensive psychophysiological and ergonomic literature supports this modeling choice: classical studies have demonstrated that psychological stress induces a sustained elevation in tonic baseline EMG activity, particularly in stabilizing muscles (Lundberg et al., 1994; Waersted et al., 1996). Similarly, increased arousal and cognitive demands have been shown to alter steady motor contractions by increasing background muscle tension and generalized co-contraction, even in muscles not strictly required for the dynamic execution of the task (Noteboom et al., 2001; Mehta and Agnew, 2012). To provide a rigorous methodological foundation for the proposed framework, we implemented a bio-inspired simulation representing center-out point-to-point tasks across eight planar directions, utilizing an eight-muscle model driven by three fundamental synergies. The simulation parameters were specifically tuned to reflect plausible physiological conditions and to test the robustness of the Augmented Reconstruction Index. In this model, the spatial synergy matrices W_ground_truth_ were unit-normalized to ensure that any observed fluctuations in Electromyographic (EMG) amplitude were strictly attributable to modifications in the temporal drive rather than structural scaling. Activations c(t) were modeled as biphasic bursts typical of fast point-to-point movements. In the present proof-of-principle simulation, both the latent contextual variable Z and the sensitivity vector β were assumed known by construction. This choice was adopted to isolate the identifiability problem associated with unconstrained decomposition methods, rather than to implement a complete inference pipeline for contextual factors. The magnitude of Z was 0.6, coupled with a sensitivity vector β set at (0.175, 0.125, 0.15)—to simulate a scenario where internal stressors or cognitive loads do not merely inject stochastic noise but systematically shift the baseline excitability of specific motor modules. Furthermore, the baseline temporal profiles c0 were modeled as asymmetric biphasic bursts to represent the acceleration and deceleration phases of reaching, incorporating a 1% stochastic gain variation to mimic natural neuromuscular jitter. The interpretation of the results derived from this simulation highlights the critical divergence between standard decomposition techniques and the ARI approach. While both standard Non-negative Matrix Factorization (NMF, Lee and Seung, 1999) and the ARI framework typically achieve high reconstruction quality, often exceeding 99% of the reconstruction R^2^ their physiological validity differs significantly. The purpose of this comparison was to evaluate whether contextual variance would be absorbed as an apparent structural modification of the spatial synergies (standard NMF) or explicitly represented as a modulation of temporal recruitment while preserving the original motor structure (ARI framework). However, it achieves this mathematical optimization by distorting the extracted spatial matrix (W_extracted_ ≠ W_ground_truth_) to absorb the contextual offset, even in this context of low noise and β. This phenomenon—which we term structural drift—creates a systematic artifact: context-driven tonic changes are misinterpreted as a fundamental reorganization of the underlying motor coordination strategy. Conversely, the proposed ARI framework employs a constrained approach. Specifically, the ARI analysis constrained the spatial synergies to the baseline estimate (W ≈ W_ground_truth_) and modeled the contextual contribution as an explicit additive modulation of the temporal coefficients through the known term βZ. By locking the spatial synergies to their baseline structure (W_fixed_ = W_ground_truth_) and explicitly modeling the residual variance through the contextual vector Z, the ARI achieves a high reconstruction accuracy (*R*^2^ = 0.9983). Standard unconstrained NMF achieved a marginally higher overall reconstruction (*R*^2^ = 0.9989). This mathematical behavior is entirely expected: because unconstrained NMF possesses more degrees of freedom (freely optimizing both W and c), it will always fit the training data equally well or better than a constrained model. However, NMF achieves this higher R^2^ through overfitting—specifically, by inappropriately distorting the spatial synergies (structural drift) to absorb the contextual variance. ARI prevents this coefficient pollution by sacrificing a negligible fraction of mathematical fit to preserve physiological validity. Crucially, the positive ΔR^2^_context_ discussed in our analytical framework (section 3.4, Equation 5) and Prediction 1 does not refer to a comparison between ARI and unconstrained NMF. Rather, it compares the ARI model R^2^_motor+context_ against a purely motor constrained baseline (R^2^_motor_ calculated using W_ground_truth_ but lacking the β⋅Z contextual modulation). Under this correct comparative baseline, the inclusion of the contextual term strictly reduces the residual reconstruction error. Finally, while the extremely low noise in this proof-of-concept simulation creates a ceiling effect (R^2^> 99% for both methods), in real-world human data (where typical R^2^ is around 90%), the inability of constrained models to handle context may force unconstrained NMF to artificially extract an additional (k+1) synergy to account for the unexplained variance. The ARI framework circumvents this by handling the variance via the temporal contextual term. Figure 2 shows the results of the simulation. To visually demonstrate this physiological realism, the simulated EMG traces for all channels and directions are depicted in Figure 3. Following established methodological paradigms for biphasic synergy activation (d’Avella et al., 2006), a canonical task-oriented profile was implemented. The figure contrasts the pure motor baseline with the contextualized condition.

### 3.7 On the reconstruction of the ground truth with ARI

This simulation highlights a critical limitation in traditional synergy analysis: a high R^2^ in standard NMF is not a definitive guarantee of physiological validity, as the model’s degrees of freedom allow it to easily overfit contextual noise, leading to “coefficient pollution.” Importantly, the present implementation should be interpreted as an illustrative proof-of-principle. In future experimental applications, the latent contextual variable Z would be estimated from physiological and/or psychometric measurements, while the sensitivity coefficients β would be statistically inferred from the observed data. In fact, a natural question regarding ecological applications is how the ARI framework can estimate the spatial synergies W, the temporal baseline drive c_0_, and the sensitivity vector β when a pure “motor-only” baseline is unavailable. Because the contextual variable Z is empirically known from physiological sensors, the simultaneous extraction of the remaining variables translates into a Constrained Non-negative Matrix Factorization problem. Theoretically, this can be solved using an Alternating Non-negative Least Squares (ANLS) optimization scheme, where the algorithm iteratively updates W, c_0_, and β while maintaining non-negativity constraints. Developing and benchmarking this specific numerical solver represents the next computational step for this framework and falls beyond the scope of the present Perspective. However, from an experimental standpoint, we recommend including a brief calibration or familiarization block in the study design whenever possible. Performing the motor task under minimal cognitive load and neutral conditions provides a highly reliable initialization matrix for W. This functional baseline anchors the subsequent ARI extraction during highly contextualized blocks, preventing local minima during optimization and further ensuring that contextual variance is properly isolated within the temporal domain. The proposed ARI has the potential to provide superior explanatory power by mathematically decoupling stable motor primitives from dynamic internal states. Consequently, it reduces the risk of false identification of synergy reorganization in scenarios where only contextual modulation of a stable primitive is occurring.

Crucially, a conventional workaround to address unexplained variance in standard NMF is to simply increase the number of extracted synergies (k). However, within the spatial synergy hypothesis, this approach lacks physiological justification for transient contextual modulations. The spatial matrix W is theorized to represent stable neuromuscular modules. A sudden shift in cognitive load or affective state modulates the central drive but does not instantaneously create a new anatomical primitive. If standard NMF is unconstrained and forced to extract k+1 synergies to account for the contextualized dataset, the algorithm will mathematically isolate the tonic offset (induced by β⋅Z) into a supernumerary, artificial module. While this strategy might increase the total variance explained, it yields a “further synergy”—a mathematical artifact that merges the biological motor structure with context-dependent noise, thereby destroying the neurophysiological interpretability of the decomposition. The ARI framework circumvents this issue: by maintaining the original dimensionality k and locking the spatial structure to the baseline primitives, it safely confines the state-dependent fluctuations to the temporal domain, explicitly modeling the variance without polluting the fundamental motor vocabulary.

## 4 Discussion

The present work proposes a conceptual and analytical extension of the muscle synergy framework, in which motor coordination is interpreted as the emergent outcome of interactions between neural, cognitive, affective, and experiential processes. By integrating these dimensions into a unified architecture and introducing a quantitative approach to capture their contribution, this framework aims to move beyond a strictly mechanistic interpretation of muscle synergies toward a context-sensitive account of motor control. It is important to clarify that providing an exhaustive computational model of the cognitive, psychological, and experiential factors that influence motor output is beyond the scope of this paper. We acknowledge that modeling these domains involves a high degree of complexity, as they emerge from non-linear interactions within the central nervous system that are not yet fully understood. Our primary objective is not to define the inner deterministic nature of these factors, but rather to provide a framework (ARI) that formally accounts for their existence during the synergy extraction process. Even in its simplest implementation—such as using a linear regression or a correlation analysis to estimate the weight of contextual variables on motor primitives—the ARI framework represents a step forward compared to traditional NMF. By explicitly including these factors, we avoid the potentially limiting interpretation of muscle synergies as purely mechanistic units, ensuring that non-motor variance is recognized and decoupled rather than ignored or misinterpreted as structural motor noise.

### 4.1 Revisiting the nature of muscle synergies

A central implication of this work is a reframing of muscle synergies from motor primitives to context-dependent coordination patterns. While the classical interpretation has emphasized the stability and neural origin of synergy structure, our results suggest that at least part of the variability observed across subjects and conditions may reflect systematic modulation by higher-level processes rather than noise or measurement error. Importantly, this perspective does not contradict the existence of low-dimensional organization in motor control, but rather refines its interpretation. The decomposition EMG = c(t)⋅ W remains a valid and useful representation; however, its components may carry different functional meanings. In particular, the distinction between spatial modules (W) and temporal activations c(t) provides a natural locus for integrating contextual influences. Within the proposed framework, higher-level variables are hypothesized to primarily modulate the temporal structure of activation, suggesting that variability in c(t) may encode information about cognitive, affective, and experiential states. Future iterations of this framework should investigate whether specific clusters of Z variables (e.g., high-intensity emotional stress) act as triggers for the reconfiguration of W, moving beyond the “fixed-primitive” paradigm.

### 4.2 From unexplained variance to structured variability

A second key contribution concerns the interpretation of variability in motor data. In traditional analyses, variance not captured by the synergy model is often treated as noise or residual error. By explicitly incorporating contextual variables into the modeling process, we show that part of this variability can be reinterpreted as structured and meaningful, reflecting differences in internal state across trials or individuals. Proposed Augmented Reconstruction Index (ARI) formalizes this idea by explicitly separating the variance explained by the motor structure from the additional contribution of contextual factors. Rather than introducing a novel scalar metric, ARI provides an interpretable decomposition of explained variance into complementary components, enabling a more precise assessment of how internal state variables contribute to motor output. While preliminary in its formulation, this approach provides a principled way to assess the relative contribution of motor structure and context-dependent modulation. More broadly, it opens the possibility of decomposing motor variability into distinct components associated with neural organization, internal state, and their interaction. Unlike simpler linear models, the use of latent factors for Z allows for a more physiologically plausible representation of the subject’s state. By extracting common variance from multiple sensors, the model becomes less sensitive to the noise of a single measure (e.g., a movement artifact in the EDA signal) and more focused on the underlying biological construct (e.g., “Stress Level” or “Cognitive Effort”). This increases the interpretability of the β coefficients, which now represent the sensitivity of motor synergies to well-defined internal dimensions.

### 4.3 Implications for experimental design and interpretation

The framework has several methodological implications for the study of motor control. First, it highlights the importance of measuring and reporting cognitive and affective variables alongside motor data. Even minimal assessments of task understanding, strategy, or emotional state may help explain differences that would otherwise be attributed to noise. Second, it suggests that inter-subject variability should not be treated solely as a nuisance, but as a potential source of information about underlying control processes. Differences in synergy structure or activation patterns may reflect systematic differences in internal models, prior experience, or affective state. Third, the framework encourages a shift from static to dynamic analyses of behavior. By considering learning trajectories and feedback loops, motor performance can be studied as an evolving process within a cognitive–motor state space, rather than as a fixed outcome.

#### 4.3.1 Basic research

From a basic research perspective, the proposed framework has important implications for the design and interpretation of motor control experiments. By explicitly accounting for cognitive, affective, and experiential variables, it challenges the common assumption that unexplained variability primarily reflects noise or measurement error. Instead, part of this variability can be reinterpreted as structured and condition-dependent. This shift suggests that experimental paradigms should incorporate minimal assessments of internal state—such as task understanding, cognitive load, or affective condition—and, when possible, manipulate these factors systematically. Moreover, inter-subject variability, often treated as a nuisance, becomes an informative signal that can reveal differences in internal models and control strategies. As a result, studies based solely on averaged synergy structures may overlook meaningful dimensions of motor organization, whereas approaches integrating contextual variables can provide a more complete and mechanistically grounded account of motor behavior.

#### 4.3.2 Rehabilitation

In rehabilitation contexts, the framework suggests a shift from a purely motor-centric view of impairment toward a more integrated interpretation of motor dysfunction. Alterations in muscle synergies are typically attributed to neural damage or biomechanical constraints; however, the present approach highlights that cognitive deficits, affective state (e.g., anxiety, motivation), and prior experience can also significantly influence motor coordination. This has direct implications for both assessment and intervention. Clinically, incorporating simple measures of cognitive and affective state may improve the interpretation of patient-specific motor patterns and reduce misattribution of variability to impairment alone. From an intervention standpoint, rehabilitation protocols could be enhanced by targeting not only motor execution but also task understanding, engagement, and emotional regulation. For instance, improving task internalization or reducing anxiety may lead to measurable changes in synergy recruitment and motor variability. More broadly, this perspective supports the development of personalized rehabilitation strategies in which motor training is coupled with cognitive and affective modulation, ultimately promoting more robust and transferable recovery of function. In human-robot interaction, ARI allows an exoskeleton control system to not confuse user stress with a change in motor intention.

### 4.4 Spatial vs. temporal effects and the limits of W invariance

A foundational assumption of the current ARI framework implementation is the structural invariance of the spatial synergy matrix W. It is critical to delimit this choice within the framework of the standard spatial muscle synergy model, where W represents time-invariant muscle weightings and c(t) denotes time-varying activations. While a comprehensive evaluation of spatial versus space-time synergy models is beyond the scope of this perspective, the neurophysiological rationale for fixing W during transient contextual shifts is compelling. Spatial primitives are widely considered to be coded into spinal and brainstem premotor networks. Transient affective or cognitive states—such as a sudden spike in performance anxiety or an acute increase in cognitive load—characteristically modulate the descending central drive rather than instantaneously restructuring these low-level anatomical networks. Consequently, these short-term factors are most appropriately modeled as gain modulators acting upon the temporal coefficients. Conversely, we acknowledge that the invariant-W assumption represents a first-order approximation that may not hold across all timelines. Experiential factors operating over extended timescales, such as prolonged motor learning, intensive athletic training, or chronic psychological stress, are well-known drivers of neuroplasticity. These long-term adaptations can indeed lead to the structural reorganization of the cortical and spinal maps, ultimately altering the composition of W itself. Future extensions of the ARI framework should therefore incorporate hierarchical layers where short-term states modulate temporal dynamics, while long-term experiential traits structurally reshape the spatial motor vocabulary W.

### 4.5 Relation to existing theoretical frameworks

The proposed approach is consistent with and extends several contemporary theories of brain function. In particular, it aligns with Predictive Processing in emphasizing the role of internal models and context-dependent modulation, and with Embodied Cognition in viewing motor behavior as inseparable from cognitive and bodily processes. At the same time, the framework provides a concrete link between these high-level theories and measurable motor variables, grounding abstract concepts such as prediction, prior, and internal state in observable quantities such as EMG activity and synergy structure. In this sense, it offers a bridge between cognitive neuroscience and motor control, domains that have often been studied in relative isolation.

### 4.6 Future directions

Future work should aim to empirically validate the proposed framework across different tasks, populations, and contexts. In particular, experiments that systematically manipulate cognitive load, emotional state, or prior experience could provide direct tests of the predicted effects on synergy activation patterns. From a methodological standpoint, further development of the ARI and related metrics could enable more precise decomposition of motor variability, including nonlinear interactions and time-varying effects. Integration with techniques such as state-space modeling or dynamical systems analysis may further enhance the characterization of cognitive–motor coupling. Finally, the framework may have applications beyond basic research, including motor rehabilitation, human–robot interaction, and performance optimization, where accounting for internal state could improve both interpretation and intervention.

### 4.7 Testable predictions and falsifiable hypotheses

A key requirement for the proposed framework is that it generates empirically testable and falsifiable predictions. While the present work is primarily conceptual and supported by a proof-of-principle simulation, it leads to a set of concrete hypotheses that can be directly evaluated in experimental settings. These predictions follow from the central assumption that a portion of the variability observed in muscle synergy structure reflects systematic modulation by cognitive, affective, and experiential factors.

#### 4.7.1 Prediction 1: affective modulation selectively alters temporal activation patterns

If affective state acts as a global modulator of motor control, then experimentally induced stress or anxiety should produce systematic changes in the temporal activation coefficients c(t), without necessarily requiring a reorganization of the spatial synergies W. Within the proposed framework, this effect should manifest as a significant increase in the context-dependent component of explained variance (ΔR^2^_context_) while R^2^_motor_ remains relatively stable. This prediction can be tested by combining standard EMG recordings with physiological or psychometric measures of stress (e.g., HRV, EDA, or validated questionnaires).

#### 4.7.2 Prediction 2: cognitive load increases context-dependent variance

Under dual-task conditions or increased cognitive load, the precision of internal task representations is expected to decrease, leading to more variable and less stable motor execution. Within the ARI framework, this should result in an increase in ΔR^2^_context_, reflecting a greater contribution of cognitive factors to motor variability. Importantly, standard synergy decomposition methods may attribute this variability to changes in the extracted modules, whereas the proposed framework predicts that a significant portion of it can be captured through context-dependent modulation of c(t).

#### 4.7.3 Prediction 3: prior experience shapes the structure of contextual factors

Differences in motor experience are expected to influence not only motor performance, but also the structure of the latent contextual variables Z. For instance, expert and novice participants performing the same task may exhibit comparable levels of R^2^_motor_, but differ systematically in ΔR^2^_context_, and in the composition of the latent factors extracted from cognitive–affective–experiential measures. This would suggest that part of the variability traditionally attributed to differences in motor primitives may instead reflect differences in embodied priors and internal representations.

#### 4.7.4 Prediction 4: learning reduces context-dependent variance over time

As a task becomes more familiar, the contribution of cognitive and affective factors is expected to decrease, leading to more stable and efficient motor execution. Within the proposed framework, this should manifest as a progressive reduction in ΔR^2^_context_ across trials, accompanied by increased consistency in the temporal activation patterns c(t). This prediction can be tested in longitudinal or trial-by-trial learning paradigms.

#### 4.7.5 Prediction 5: misinterpretation of structural changes under standard decomposition

In conditions where contextual factors introduce systematic offsets or modulations in motor output, standard unconstrained decomposition methods (e.g., NMF) are expected to partially absorb these effects into the spatial structure W, leading to apparent—but potentially spurious—reorganization of muscle synergies. In contrast, the ARI framework predicts that explicitly modeling contextual variables will preserve the stability of W while attributing variability to changes in c(t). This prediction directly extends the behavior observed in the toy model to experimentally testable scenarios.

These predictions provide a concrete roadmap for the empirical validation of the proposed framework. Importantly, they do not require complex or invasive experimental setups, but can be tested within standard motor control paradigms augmented with minimal assessments of cognitive and affective state. As such, the framework is not only conceptually grounded, but also experimentally actionable, even if an effort is required to model and balance the effect of contextual parameters. To prevent theoretical ambiguity, it is critical to clarify the precise relationship between the ARI framework and the principles of Predictive Processing. The ARI model does not compute an online, real-time prediction error in the canonical sense of active inference microcircuits—where descending motor commands are continuously matched against high-frequency sensory feedback at a millisecond scale. Rather, it operationalizes predictive principles at a macro-systemic level, treating cognitive, affective, and experiential variables as top-down contextual priors that parameterize and modulate the lower-level generative mechanisms of muscle synergy recruitment. Within this conceptualization, the residual variance or reconstruction discrepancy observed when applying standard NMF to contextualized datasets represents the unmodeled systemic variance that emerges when these high-level priors are omitted from the decomposition. By capturing and formalizing this discrepancy, the ARI framework explicitly quantifies how top-down states contextualize the lower-level motor vocabulary, aligning with a hierarchical and integrated view of sensorimotor control.

### 4.8 Limitations

Several limitations should be acknowledged. First, the measurement of cognitive and affective variables relies on proxies that are inherently indirect and potentially noisy. Self-report measures, in particular, may be subject to bias and limited temporal resolution. As discussed, bridging the temporal gap between the millisecond resolution of motor control and the much slower, coarser evolution of internal states requires careful experimental design, prioritizing continuous physiological tracking (e.g., autonomic signals) over invasive and fatiguing periodic questionnaires. Second, the proposed modeling framework assumes that contextual variables primarily influence the temporal activation of synergies. While this hypothesis is grounded in current evidence, it may not fully capture all forms of modulation, and in some cases higher-level processes may also affect the structure of the modules themselves. Integrating such “structural plasticity” into the ARI would require more complex longitudinal datasets, which represents a primary direction for the evolution of this modeling approach. Moreover, ARI capture instantaneous dynamics (e.g.: the stress perceived in that very moment), rather than longitudinal modifications. Third, the Augmented Reconstruction Index provides an aggregate measure of context-dependent contribution, but does not by itself identify causal relationships. Establishing causality will require carefully designed experimental manipulations and, potentially, interventions targeting specific layers. Finally, the framework introduces additional complexity into experimental design and analysis. We acknowledge that the absence of empirical validation limits the immediate applicability of the framework. However, the goal is to expose a methodological blind spot rather than provide a finalized model. While we have emphasized approaches that can be implemented with limited overhead, there is an inherent trade-off between model richness and practical feasibility. More broadly, the proposed framework remains at a conceptual level, and the analytical formulations introduced here should be interpreted as preliminary steps toward a full computational account.

Another critical consideration regards the mathematical nature of the coupling between latent contextual states and temporal activations. The present implementation assumes a linear, additive relationship, and treats the latent vector Z as a low-dimensional reduction of individual factors. In ecological human behavior, however, cognitive load, anxiety, and sensorimotor memory are highly unlikely to combine linearly. For instance, high levels of prior experience typically alter the baseline sensitivity to acute stress, suggesting the presence of significant interaction effects (Z_stress_ ⋅Z_experience_). Crucially, the conceptual architecture of the ARI framework is non-linear by design and can flexibly accommodate more sophisticated coupling structures. Future empirical applications on human subjects will necessitate replacing the first-order linear equations with non-linear mapping functions—such as sigmoidal scaling to capture physiological saturation, or non-linear state-space representations—to accurately model the threshold-dependent and multiplicative effects of psychological load on the motor drive.

Furthermore, a methodological limitation of the current proof-of-concept implementation lies in the use of Principal Component Analysis (PCA) to reduce the dimensionality of heterogeneous non-motor variables. While PCA effectively addresses multi-collinearity and enhances algorithmic stability by preventing overfitting, it inherently collapses distinct, independent internal drives into a single shared latent space ($Z$). Consequently, this approach may reduce specificity, potentially masking the individual and unique contributions of specific factors (e.g., decoupling transient performance anxiety from cognitive fatigue) on muscle synergy modulation. Future experimental applications aiming to explicitly disentangle these distinct physiological and psychological pathways should transition from an aggregate PCA approach to more granular statistical frameworks, such as structural equation modeling (SEM) or regularized multi-variable partial regression.

## 5 Conclusion

In this work, we proposed an extension of the muscle synergy framework in which motor coordination is understood as the outcome of interactions between neural, cognitive, affective, and experiential processes. By introducing a structured architecture and an accompanying analytical approach, we argued that a significant portion of variability in motor output—traditionally treated as noise—can instead be interpreted as reflecting context-dependent modulation of control. Within this perspective and coherently with the spatial synergy model, muscle synergies are viewed as fixed motor primitives, but their recruitment is seen as an emergent, context-sensitive set of activation patterns, shaped by the dynamic coupling of multiple layers of control. The proposed Augmented Reconstruction Index (ARI) provides a structured decomposition of motor variability into motor and context-dependent components, enabling a more nuanced interpretation of coordination patterns. Overall, this framework offers a principled way to integrate motor and cognitive dimensions of behavior, providing both a conceptual shift and a practical direction for future experimental and analytical work in motor control.

## Data Availability

The raw data supporting the conclusions of this article will be made available by the author upon reasonable request.
